# Global expression profiling reveals genetic programs underlying the developmental divergence between mouse and human embryogenesis

**DOI:** 10.1186/1471-2164-14-568

**Published:** 2013-08-20

**Authors:** Lu Xue, Jin-Yang Cai, Jian Ma, Zan Huang, Ming-Xiong Guo, Lie-Zhen Fu, Yun-Bo Shi, Wen-Xin Li

**Affiliations:** 1 College of Life Sciences, Wuhan University, Wuhan 430072, P.R China; 2 Institute for Medical Biology, College of Life Sciences, South-Central University for Nationalities, Wuhan 430074, P.R China; 3 Section on Molecular Morphogenesis, Program in Cellular Regulation and Metabolism, NICHD, National Institutes of Health, Bethesda, MD 20892, USA

**Keywords:** Microarray, Mouse embryogenesis, Human embryogenesis, Organogenesis, Evolution, Protein interaction network

## Abstract

**Background:**

Mouse has served as an excellent model for studying human development and diseases due to its similarity to human. Advances in transgenic and knockout studies in mouse have dramatically strengthened the use of this model and significantly improved our understanding of gene function during development in the past few decades. More recently, global gene expression analyses have revealed novel features in early embryogenesis up to gastrulation stages and have indeed provided molecular evidence supporting the conservation in early development in human and mouse. On the other hand, little information is known about the gene regulatory networks governing the subsequent organogenesis. Importantly, mouse and human development diverges during organogenesis. For instance, the mouse embryo is born around the end of organogenesis while in human the subsequent fetal period of ongoing growth and maturation of most organs spans more than 2/3 of human embryogenesis. While two recent studies reported the gene expression profiles during human organogenesis, no global gene expression analysis had been done for mouse organogenesis.

**Results:**

Here we report a detailed analysis of the global gene expression profiles from egg to the end of organogenesis in mouse. Our studies have revealed distinct temporal regulation patterns for genes belonging to different functional (Gene Ontology or GO) categories that support their roles during organogenesis. More importantly, comparative analyses identify both conserved and divergent gene regulation programs in mouse and human organogenesis, with the latter likely responsible for the developmental divergence between the two species, and further suggest a novel developmental strategy during vertebrate evolution.

**Conclusions:**

We have reported here the first genome-wide gene expression analysis of the entire mouse embryogenesis and compared the transcriptome atlas during mouse and human embryogenesis. Given our earlier observation that genes function in a given process tends to be developmentally co-regulated during organogenesis, our microarray data here should help to identify genes associated with mouse development and/or infer the developmental functions of unknown genes. In addition, our study might be useful for invesgtigating the molecular basis of vertebrate evolution.

## Background

For over 50 years, the mouse (Mus musculus) has played a prominent role for studying mammalian development. Studies in mouse have led to enormous progresses in our understanding of early human development, which are difficult to study directly due to the difficulty in obtaining suitable samples ethically and technically [1,2]. The identification of genes and the signaling pathways involved in mouse embryogenesis have helped us to better understand embryonic development in mammals, including fertilization, morulation, gastrulation, and organogenesis [3]. Understanding the molecular mechanisms governing mouse embryogenesis is also important for translational and clinical research in diverse areas such as reproductive biology, regenerative medicine, and genetic therapy.

**Figure 1:**
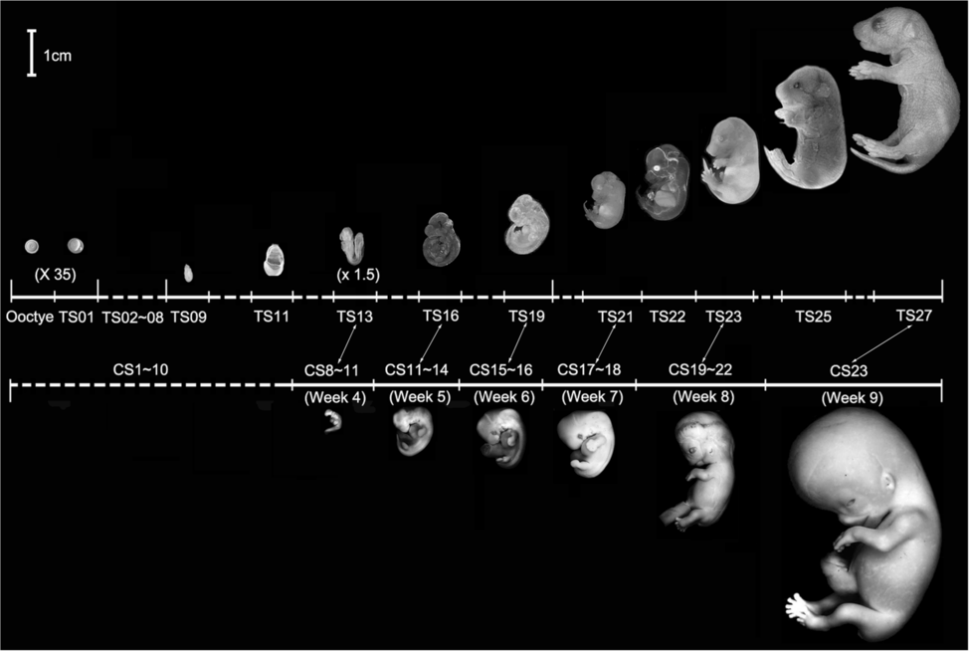
**Morphological comparisons of mouse and human embryo development.** Mouse embryonic stages (Theiler stages or TS) are based on somite number and characteristics [26]. There are 28 TS stages from the fertilization to birth, which is about 20 days post conception (dpc). Only 11 stages are shown. Human embryo stages were described by the Carnegie Institution of Washington, which are based on the developmental structures, not by size or the number of days of development [27]. The 23 Carnegie stages (CS) only covers the first 60 days of human embryo development, thereafter that the term embryo is replaced with fetus. 5 human embryos at 4-9^th^ week are shown here. The double headed arrows point to human and mouse embryos at similar stages of organogenesis.

The advent of microarray analysis has allowed systematic analysis of gene expression changes during development. Elucidating the transcriptomes of successive developmental stages in different animal species is critical for understanding the developmental mechanism and revealing the conservation and diversification at molecular level among species during embryogenesis. A number of reports on genome-wide expression profiles for different developmental periods during human and mouse embryogenesis have been published, which have provided valuable information on the gene and the molecular network underlining morphological changes during these periods [4-24] These earlier studies so far focused mainly on oocytes and preimplantation embryos in mouse and revealed a number of similarities between mouse and human gene expression profiles. This perhaps was not surprising given the similarities between the two species at early stages of development (Figure 1, Additional file 1A). On the other hand, the arguably more critical period of embryogenesis is the period of organogenesis, when most significant morphological differences occur between human and mouse (Figure 1) [25]. One of the most obvious differences between mouse and human embryogenesis is the time of birth. The mouse embryo is born almost immediately after all the organs develop (around TS27-TS28, 19–20 days post conception, Figure 1). On the other hand, at the end of organogenesis (CS23, corresponding to TS26/27, Figure 1), the human embryo has a disproportionally large head relative to the whole body and other organs. The embryo continues to stay in the uterus for a few more months, a period termed as the fetal stage. During this stage, many organs continue to grow and eventually develop into their proper sizes for birth. The underlying molecular basis for this developmental divergence remains unknown as there have been essentially no global gene expression data available for mammalian embryos past the peri-implantation period. Interestingly, two recent studies on the global gene expression profiles during human organogenesis (up to 9 weeks of human gestation) revealed very interesting and informative information on the gene regulation networks governing organogenesis, distinct from those involved in early embryogenesis, which utilizes many maternal genes [7,18,25].

Here we report the first genome-wide study of the gene expression profiles of mouse embryos spanning all stages of mouse development. Our analyses demonstrate conservations in the gene regulation networks underlying early embryogenesis in mouse and human but also reveal distinct molecular pathways that underlie the morphological and functional divergences during late organogenesis, leading to the birth at the end of organogenesis in mouse while several additional months of gestation in human.

## Results and discussion

### Dynamic changes in the transcriptome throughout mouse embryogenesis

The Theiler stages are defined on the basis of anatomical features during mouse embryogenesis [26], which serve as references for comparative study of embryogenesis among different species. To determine the developmental gene expression profiles, mouse C57BL/6 J oocytes (eggs), TS01, and embryos at 11 Theiler stages [26] that covers the entire embryo development period were collected (Figure 1). To minimize the individual difference during embryonic development, each biological replicate included at least 500 eggs or 20 embryos and three biological replicates of each stage were subjected to Affymetrix Mouse 430 expression microarray analysis. In addition, a heatmap analysis showed that the data from different biological replicates at each time point were reproducible (Additional file 1B). Finally, RT-PCR analysis of many genes on an independent RNA set confirmed microarray findings (described below).

For the microarray analysis, a transcript was scored as “detected” or “expressed” if significant signal was detected in two or three individual microarrays of the three independent samples [28]. Overall, 27,857 out of the 39,015 transcripts on the microarray were detected at least in one of the eleven stages (Additional file 2).

**Figure 2:**
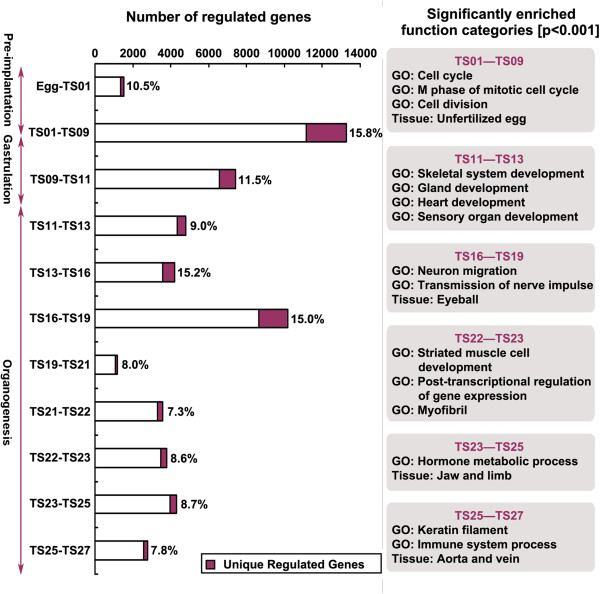
**Early embryogenesis involves many more regulated genes than late organogenesis during mouse development.** The gene expression levels were compared between each pair of adjacent developmental stages to identify developmentally regulated genes. The total number of the genes regulated between the two stages (p < 0.01) were shown as a horizontal column (open and purple shaded columns). The number of genes whose expression levels were changed only between the two indicated stages, thus termed unique regulated genes, was shown as the purple shaded portion of the columns and its percentage in the total regulated genes was shown next to the column. Some enriched function categories (Gene Ontology or GO) for each group of uniquely regulated genes are shown on the right.

The goal of our study is to determine 1) whether and how transcriptome differs among different stages of mouse embryogenesis, and 2) how the changes in transcriptome correlate with the embryogenesis. To address these questions, we compared the expression profiles between adjacent stages during development. We observed that the extent of the changes in transcriptome, i.e., the number of genes that exhibited differential expression levels between two adjacent stages during mouse embryogenesis, correlated well with gross morphological changes of the embryo (Figure 2), that is, the more different the morphologies between two adjacent stages, the larger the number of genes whose expression was altered (Figure 2). For example, when embryos at the preimplanting zygote stage (TS01) developed into early gastrula stage (TS09), the individual zygotes or single cell fertilized eggs changed dramatically through zygotic division into differentiated gastrula embryos with three germinal layers [29]. Such drastic changes in morphology were accompanied by the largest number (about 13,000) of genes with different expression levels (Figure 2). A similar large number (about 10,000) of genes with altered expression levels were detected between TS16 and TS19 when both stages are at the early stages of organogenesis and the mouse embryo grew and changed rapidly as the somites formed during early organogenesis (Figure 2). In contrast, there were relatively few genes with significantly altered expression between unfertilized eggs and TS01 or between TS19 and TS21, when the gross morphology change was relatively minor (Figure 2). These results suggest that the extent of the morphological changes is well correlated with the number of genes whose expression is altered.

As the developmental changes between two adjacent stages vary during embryogenesis and may thus involve genes whose expression is only altered between these two adjacent stages, we examined the number of genes that were regulated only between any adjacent two stages, which we termed “uniquely regulated genes” (Additional file 2). The numbers of such genes and the percentage of such genes in the total number of regulated genes between the two adjacent stages were shown in Figure 2 (shown as horizontal columns and the numbers in%, respectively). Again, we found that the number as well as the percentage of uniquely regulated genes correlated with the extent of the morphological changes. Interestingly, even in the percentage term, the more dramatic the morphological changes were between the two stages, the higher the percentage of the total regulated genes were uniquely regulated between two adjacent stages, suggesting that more dramatic morphological changes involves a higher proportion of genes specifically altered for the developmental processes. One exception is between TS13-TS16, which involved a very high percentage of uniquely regulated genes, even though the total numbers of regulated genes were fairly small. This may be due to the fact that the embryo is beginning a major shift in development, the onset of organogenesis.

To investigate the possible role of the uniquely regulated genes, we identified the significantly enriched Gene Ontology (GO) categories among the uniquely regulated genes. Here we observed that the enriched GO categories closely correlated with the specific morphological changes between two adjacent stages. For example, cell division was most active at TS01 ~ TS09 during mouse embryogenesis [30]. Consistently, cell division- and mitosis-related genes were highly enriched in the uniquely regulated genes between TS01 and TS09 (Figure 2). Likewise, between TS11 and TS13 as the embryo transitioned from gastrulation to organogenesis, genes belonging to the GO categories such as skeletal and heart development were enriched among the uniquely regulated (Figure 2). In addition, between TS16 and TS19 when the nerve system develops [31,32], a number of nerve development related GO categories such as neuron migration and transmission of nerve impulse were significantly enriched among the unique regulated genes (Figure 2). Even after TS19 when organogenesis was almost completed, the function of the uniquely regulated genes remained correlated with the landmarks of the developmental changes, albeit the number of the uniquely regulated genes was lower than those at earlier stages. For example, at developmental stage TS22 ~ 23, muscle tissues in some organs such as the tongue and the esophageal passage developed extensively. This was accompanied by the changes in many myofibril-specific and other striated muscle cell development-associated genes among the unique regulated genes for the stages [33] (Figure 2). Likewise, as the limb and nails become visible around stages TS23 to TS25, the genes related to jaw and limb development were enriched among the unique regulated genes. These and the similar associations at other stages (Figure 2) suggest that the regulation in the expression of the uniquely regulated genes temporally correlate with the specific morphological changes and unique developmental events at these stages, supporting important roles of these uniquely regulated genes at these developmental stages.

### Major temporal regulation patterns correlate with specific developmental processes during mouse embryogenesis

**Figure 3:**
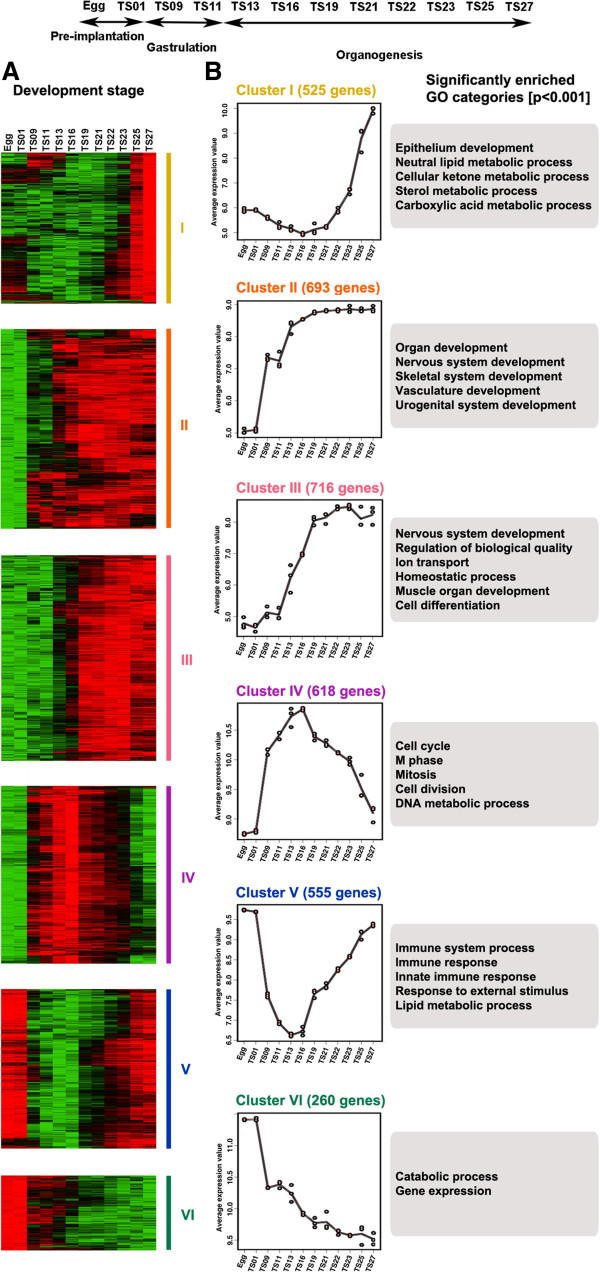
**Different temporal regulation clusters are enriched with genes of distinct functional GO categories.** The 11,458 regulated genes were divided into 20 clusters, Cluster I to XX (Additional file 3) (Additional file 4). Then the 20 clusters were divided into six groups referred to as group 1 to 6 (Additional file 4). One representative cluster from each of the six groups was shown here as Cluster I to VI, respectively. **(A)** Heat map (green to red: low to high levels of expression) showing the expression of the individual genes in Cluster I-VI. **(B)** Expression of the genes at different stages of development. Solid lines are drawn joining the average value of gene expression at each stage in the cluster. The dots reflect actual expression values. Some significantly enriched biological processes/categories for each cluster are shown in the grey box on the right.

It is well known that genes functioning in a given biological process tend to be coordinately regulated. To identify groups of genes that likely participate together in different processes during mouse development, we used the maSigPro procedure [34] to identify co-regulated gene groups across the 11 Theiler stages. Among the 27,857 genes that were detectable at least in one of the 11 stages, 11,458 genes were significantly regulated during embryogenesis. To determine the gene regulation pathways that control mouse embryogenesis, we grouped the 11,458 regulated genes into 20 clusters according to their expression patterns by means of Serial Expression Analysis (Additional file 3). We further grouped the 20 clusters into six groups of related clusters, referred to as group 1 to 6 (Additional file 4), based on the similarity in the temporal appearance of lowest and highest expression levels for each cluster. Out of each group, we chose a cluster representing the most dominant temporal developmental patterns, shown in Figure 3 as Cluster I to VI, corresponding to group 1 to 6, respectively (Additional file 4).

To validate microarray findings, we selected a few dozens of genes within different clusters for quantitative RT-PCR (qRT-PCR) analysis on an independent set of RNA samples. The results showed that the expression patterns obtained from the RT-PCR analysis agreed with those from the microarray (Additional file 1C).

To determine the potential functions of the genes within individual clusters, the genes within each of the 6 clusters above were analyzed by using DAVID tools [35] to determine their significantly enriched functional or regulatory features, including GO categories, the associated biological pathways, and tissue distribution preferences. Among the six clusters, clusters with “up-regulated” expression patterns, i.e., Clusters I, II, and III, had the lowest expression levels at the beginning of embryogenesis and highest expression toward the end of development (Figure 3). However, significant differences existed among them at different development stages, consequently leading to distinct functional categories of genes enriched among them. Genes in Cluster I had a low expression level through pre-implantation, gastrulation, to early organogenesis, but then had their expression rise sharply near the end of organogenesis (Figure 3). This cluster was enriched with genes affiliated with epithelium development (FDR = 0.19) and metabolic processes such as neutral lipid metabolic process (FDR = 0) and carboxylic metabolic process (FDR = 0) (Additional file 5). The upregulation of epithelial tissue-specific genes at the late stages agrees well with the organ development and maturation during this period. A typical example is *keratin 13*, which is involved in the development of the circumvallate papillae during the formation of tongue [36]. In addition, genes in some metabolic process-related GO categories were enriched in most gene expression clusters and the metabolic process-related genes in cluster I were implied in liver functions. For example, *sterol* is mainly synthesized in the liver; cellular ketone metabolic process and neutral lipid metabolic process are also important for lipid metabolism in the liver. These findings agreed well with the facts that the mouse liver has a critical role in blood supply at the early stages (TS18-19) and begins to function in metabolism starting from TS21 [3]. Thus, the genes identified in cluster I may play important roles in liver and epithelium development.

The expression of genes in Cluster II had a first sharp rise between pre-implantation and gastrulation, and a second rise between gastrulation and organogenesis (Figure 3). These two developmental transitions affect the entire embryo. Not surprisingly, genes in the Cluster II were not clearly enriched for genes of particular organ systems. Instead, the cluster was enriched with GO categories for many common development events like organ development (FDR = 0), skeletal system development (FDR = 0), nervous system development (FDR = 0) and urogenital system development (FDR = 0), etc. (Additional file 6). Many well-studied genes known to be important for development such as *Gli2, Notch3, Foxc2*[37-39] were found in this developmental cluster.

Compared with Cluster II, Cluster III is more likely a specialized “organogenesis” cluster since it contained groups of genes that had low levels of expression at pre-implantation and gastrulation stages but dramatically increased their expression at the beginning of organogenesis. Interestingly, there was little overlap in the enriched biological function (GO) categories between these two clusters. Cluster III was heavily enriched with genes in GO categories associated with homeostatic process (FDR = 0) and ion transport (FDR = 0) although there was also enrichment for genes in nervous system development (FDR = 0) and muscle organ development (FDR = 0.01) (Figure 3, Additional file 7). This suggests that genes that are involved only during organogenesis are distinct from those participating in both gastrulation and organogenesis.

The expression patterns for Clusters IV and V were “arch-shaped” and were mirror image of each other, with the genes in Cluster IV having highest expression levels at TS16 while those in Cluster V having the lowest expression levels at TS16 (Figure 3). Cluster IV was highly enriched with genes in cell cycle (FDR = 0), M phase (FDR = 0), mitosis (FDR = 0) and cell division (FDR = 0) (Figure 3, Additional file 8), in agreement with the active cell proliferation during TS11 ~ 13 [30]. In contrast, genes in Cluster V had the highest expression levels in egg, and then a precipitous drop in gene expression during the first half of the embryogenesis and finally a rise during the second half. In general, the genes with high levels of expression in the egg are regarded as maternal genes. Interestingly, the most noticeable, highly enriched GO categories were immune system process (FDR = 0), immune response (FDR = 0) and lipid metabolic process (FDR = 0) (Figure 3, Additional file 9). It is palpable to understand that the immune system-associated genes are significantly upregulated at the late stages of mouse development when the mouse immune system is developed and begins to function. The abundance of these genes in the mouse eggs implies the involvement of these genes in innate immune system. These findings may provide new clues for studying the development of innate immune system.

Cluster VI was the only “down-regulated” cluster among all major clusters, we expected to see that this cluster be enriched with genes in GO categories related to “pluripotency” because of the prevailing understanding that stem cell pluripotency gradually disappears in parallel with progressive cell differentiation during embryo development [40]. Surprisingly, the most noticeably enriched GO categories in Cluster VI were generic ones such as catabolic process (FDR = 0) and gene expression (FDR = 0) (Additional file 10). This may reflect the fact that the major processes associated with early embryogenesis is yolk utilization and the genes associated with such metabolic processes are no longer needed subsequently.

Thus, the different gene regulation clusters are enriched with genes that likely function together to facilitate the specific developmental processes at different stages of mouse embryonic development.

### Gene regulation during mouse organogenesis

As discussed above, mouse and human embryogenesis share extensive similarities but also have distinct differences. In particular, mouse and human development differs during organogenesis such as tail and sensory organ development. We have previous determined the gene regulation profiles at the 4 ~ 9^th^ weeks of human embryogenesis, covering the organogenesis period [18]. These stages correspond to mouse organogenesis at TS13-27/28 (Figure 1). To compared the human and mouse transcriptomes during organogenesis, we first analyzed the gene regulation profiles during mouse organogenesis.

**Figure 4:**
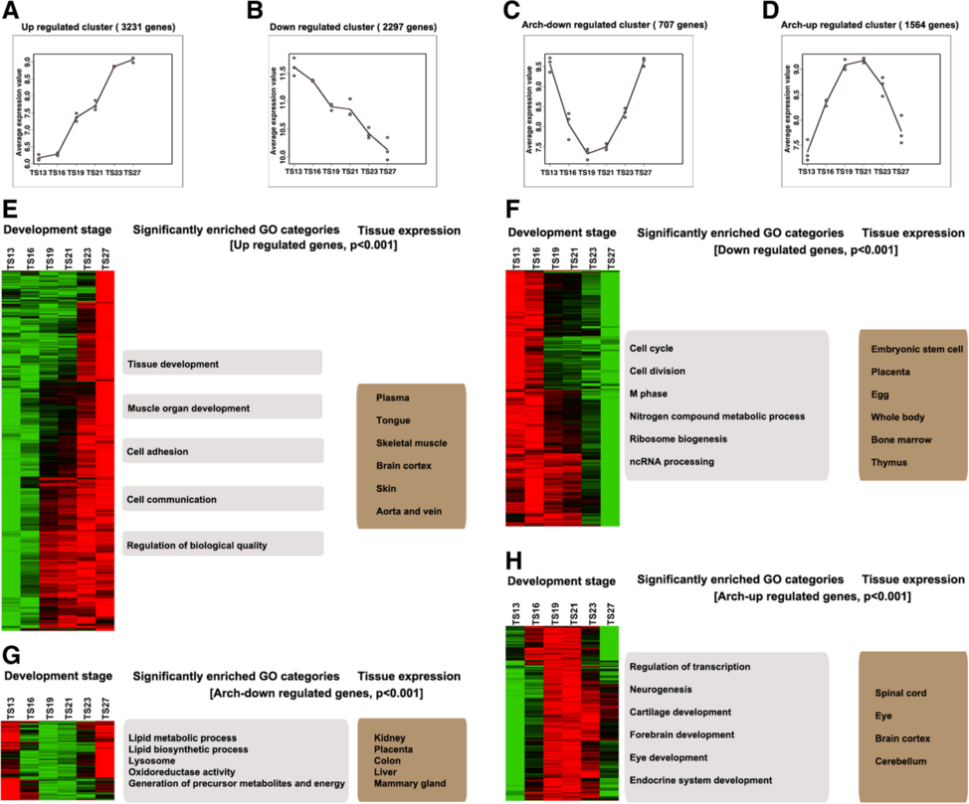
**Major temporal expression patterns during mouse organogenesis. (A)-(D)** Genes that were significantly regulated during organogenesis (between stages TS13-TS27) were clustered and the four major patterns with genes that were gradually up-, down-, arch-down-, and arch-up-regulated are shown, respectively. **(E)-(H)** GO analysis were carried out on the genes belonging to the 4 clusters shown in A-D and the corresponding biological categories highly enrichments (p < 0.001) in each of the four gene patterns are shown in E-H, respectively. In addition, the tissues where these genes are preferentially expressed are also shown for each pattern (p < 0.001), indicating distinct tissue specificities.

Analysis of the transcriptome of mouse embryos at 6 stages from TS13 to TS27, namely TS13, 16, 19, 21, 23 and 27, revealed that 8,521 genes were significantly regulated during this developmental period. Most of these genes could be clustered into 4 temporal groups (Figure 4) (Additional file 11). Group I represented the up-regulated gene clusters in which all the genes were gradually up-regulated from TS13 (Figure 4A); group II represented the down-regulated gene clusters (Figure 4B) in which all the gene were gradually down-regulated from TS13; group III represented the arch-down regulated gene clusters in which all the genes were downregulated from TS13 and after reaching a trough at TS19-TS21, were upregulated again (Figure 4C); the group IV represented the arch-up regulated gene cluster in which all the genes were first upregulated starting TS13 and after reaching a peak at TS19-TS21, were downregulated again (Figure 4D).

To investigate the potential roles of these different gene clusters in organogenesis, we determined the enriched GO categories and tissue preference of the genes in the clusters by using the methods of Huang da et al. [35]. The enriched GO categories in the genes of group I included tissue development and muscle organ development, etc. They were preferentially expressed in skeletal muscle, brain cortex, skin, etc., tissues that develop at late stages of organogenesis (Figure 4E) (Additional file 12). This is consistent with the potential role of genes in group I during this period. On the other hand, the enriched GO categories in the genes of group II included cell cycle and cell division (Figure 4F). Their downregulation likely reflects the broad transition from cell division during early embryogenesis to mainly cell differentiation during organogenesis. Another noticeable feature for this group was the enrichment of RNA splicing-specific genes, especially ncRNA (non-coding RNA) processing-associated genes. While the functional significance remains to be determined, many genes of this group were highly expressed in eggs (Additional file 13) and were among the genes in the only downregulated gene cluster (Cluster VI) during embryogenesis as described above, suggesting that the down-regulation of these genes started during early embryogenesis and continued during organogenesis.

The genes in group III were enriched with genes in a series of GO categories associated with energy generation and utilization such as lipid metabolic and lipid biosynthetic processes, transport, localization, oxidoreductase activity, and generation of precursor metabolites and energy. Data from tissue expression enrichment analysis indicated that these genes were mainly expressed in those organs involved in metabolism such liver, kidney and the mammary gland (Figure 4G) (Additional file 14). These genes are likely involved during early development in cell proliferation but not critical for differentiation during organogenesis. Toward the end of organogenesis, they are upregulated again as the organs mature to exert physiological function.

The enriched GO categories in the genes of group IV included cartilage development, forebrain development, and eye development, with the genes preferentially expressed in the corresponding tissues/organs (Figure 4H) (Additional file 15). This appears to be consistent with the fact that as the development of these organs/tissues completes, the expression of these genes are no longer needed when the embryos prepares for postnatal life.

### Conserved as well as divergent gene regulation patterns underlying mouse and human organogenesis

**Figure 5:**
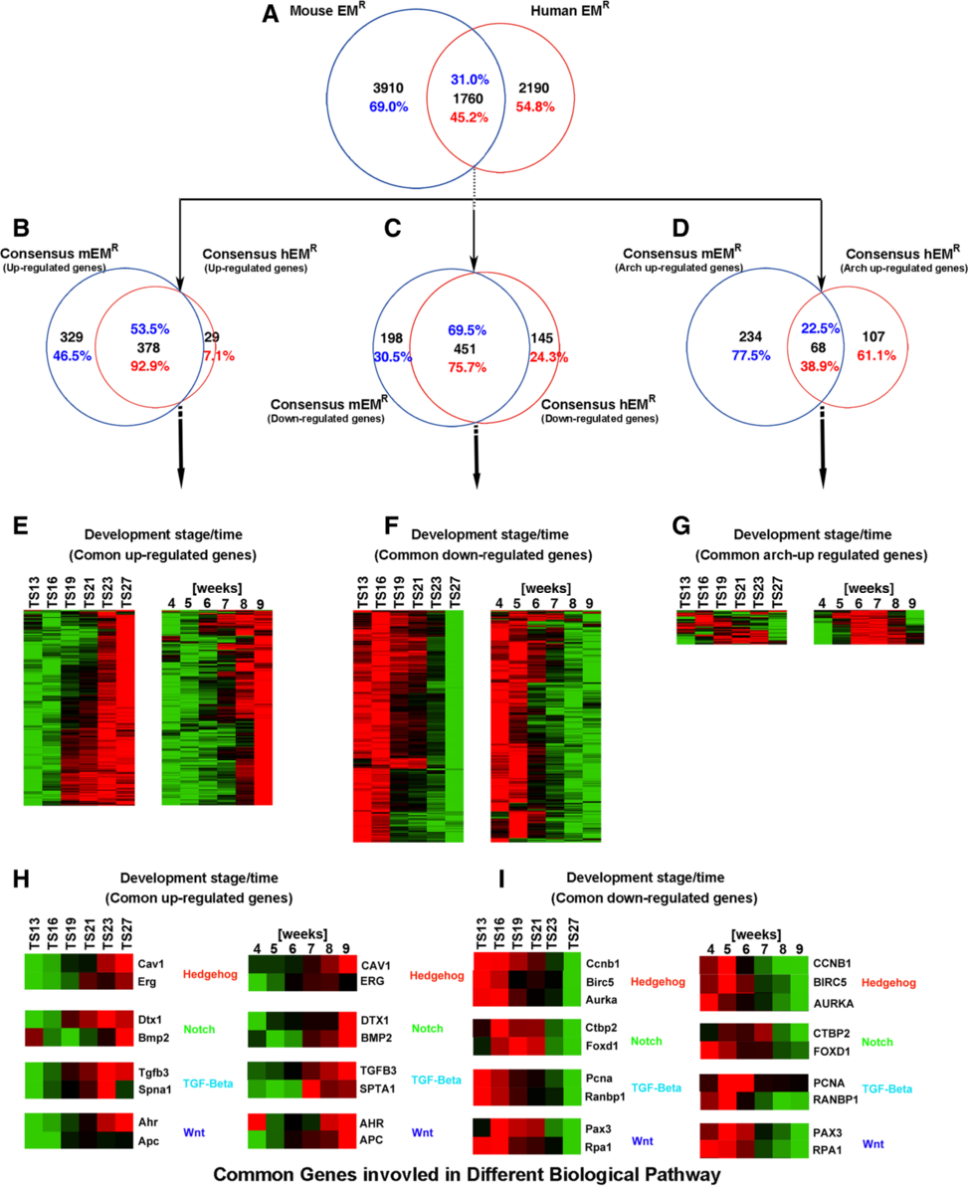
**Most of the up- and down-regulated genes are common during mouse and human organogenesis. (A)** Venn diagrams between mouse and human genes whose expression significantly changed during organogenesis. **(B-D)** Venn diagrams of the subsets of genes in panel **(A)** that were up- **(B)**, down- **(C)**, and arch-up- **(D)** regulated, respectively. **(E-G)** Heat-map expression profiles of the common genes between human and mouse in **B–D**, respectively. **(H-I)** Heat-map expression profiles of selected genes involved in 4 signaling processes that are up- **(H)** or down- **(I)** regulated similarly during organogenesis in mouse and human.

Based the most recent report of the Mouse Genome Database (MGD) [41], 5,670 out of the 8,521 mouse genes that were regulated during organogenesis have known orthologous human genes. During the corresponding period of human organogenesis, i.e., between the 4-9th week, 5,358 genes were significantly regulated [18], of which 3,950 genes have known mouse orthologs. Surprisingly, when we compared the 5,670 regulated mouse genes with the 3950 regulated human genes, we found that only 1,760 (~31%) genes were commonly regulated during organogenesis in both species (Figure 5). This ratio likely underestimate the commonly regulated genes between human and mouse since 1) many orthologous genes have not yet been annotated between mouse and human in the database and 2) there are likely other regulated genes not yet identified by the microarray studies. Nonetheless, it is much less than the 95% homology between mouse and human genome, suggesting distinct regulation of the transcriptome during mouse and human organogenesis.

Of the four major clusters of the genes regulated during mouse organogenesis, only three, groups I, II, and IV (Figure 4), were identified among the genes regulated during human organogenesis [18]. When the genes in the corresponding groups were compared, we found that 378 genes were among the common upregulated genes in mouse and human (p = 0.00), accounting for 53.5% of the genes in the mouse up-regulated gene cluster and 92.9% of the genes in the human up-regulated gene cluster, respectively (Figure 5B). Similarly, there were 451 genes were commonly downregulated in both mouse and human (p = 0.00), comprising 69.5% of the genes in the mouse down-regulated gene cluster and 75.7% of the genes in the human down-regulated gene cluster, respectively (Figure 5C). These shared upregulated or downregulated genes during organogenesis not only exhibited similar gene expression pattern (Figure 5E-F) but, more importantly, also had similar enriched GO groups as exemplified by genes in several signal transduction pathways (Figure 5H-I). Many of the commonly upregulated genes likely participated in the development of the individual organs while the shared downregulated genes were involved the cell proliferation prior to organogenesis. For example, the commonly upregulated genes included genes such as *EGFR*, which is closely associated with epidermal development [42], and *BMP2*, which is important for skeleton development [43,44]. Likewise, among the commonly downregulated genes, were *BIRC5*, *aurora A/B* and many members of cyclin family genes whose functions were significantly associated with cell cycle and cell proliferation [45-48]. They were down regulated as the embryo changed from mainly cell proliferation to organ development. Thus, these commonly regulated genes likely play critical roles for the conserved developmental processes between mouse and human.

For the third common cluster, group IV or the arch-up regulated gene cluster, there were only 68 common genes, comprised of only 38.9% of the mouse arch-up regulated gene cluster and 22.5% of the human arch-up regulated gene cluster, respectively (Figure 5D), much less than the other two groups (Figure 5B, C) (p = 0.03), suggesting that this expression pattern (Figure 5G) was not highly conserved during organogenesis. Consistently, GO analysis indicated that genes in this group might have different roles during mouse and human development. In human, the genes in this arch-up regulated gene cluster were enriched with genes associated with various metabolic processes,transcriptional regulation and eye development [18], and there was no enriched tissue expression except for genes during eye development. On the other hand, in mouse, the genes in this cluster had enriched with genes associated with the forebrain development, neurogenesis, cartilage development in additional to eye development, etc. (Additional file 15). The most noticeable common feature was that the genes associated with eye development, which is consistent with the conserved eye developmental events, such as the eye ball formation as well as pigmentation in the eyes, etc., in both mouse and human during this period.

Unlike the above three groups of genes regulated during organogenesis, there was no significant group for the arch-down regulated genes during human organogenesis [18]. It is tempting to hypothesize that genes in this cluster may have important roles in determining the developmental divergence during mouse and human organogenesis. Carefully examination revealed that many of the mouse genes in this group (arch-down or group III) (Figure 4C) were present in the group II of the downregulated gene cluster during human organogenesis (data not shown). These genes were expressed at very low levels toward the end of organogenesis in human, which contrasted sharply to their upregulation at the similar stages of mouse development (Figure 4C). As indicated above, the genes in mouse group III were enriched with genes in a series of GO categories associated with energy generation and utilization. Our finding suggests that one of the significant differences between the mouse and human development is on energy generation and consumption toward the end of organogenesis. The difference may be responsible for the major developmental difference between mouse and human, that is, the birth of the mouse embryo at the end of organogenesis vs. the extended in utero growth of the human embryo after organogenesis. The striking difference between the lengths of pregnancy in mouse and human requires different strategies for energy generation and utilization as well as other metabolic processes. During the period from stage TS13 to TS19, the mouse depends on the placenta for energy supply, just like the human embryo at the similar stages. Starting from stage TS19, as the embryonic organs associated with metabolism such as the liver gradually mature, the placenta degenerates and the energy supply and metabolite exchanges are gradually switched from the placenta to the liver. This transition prepares the fetus for their independent postnatal life upon birth [49]. Thus, the genes associated with energy metabolism in the eggs that are downregulated at the earlier stages will need to be upregulated again after TS19 when the placenta degenerates and the organs associated with energy metabolism such as the liver mature (Figure 4G). On the other hand, the human embryo of similar stages and even after organogenesis still uses the placenta for energy and metabolite supply, so the genes associated with such functions do not need to be upregulated at late organogenesis stages.

Thus, by comparing to the recently published gene expression profiles during human organogenesis, we identified 1,760 commonly regulated genes during mouse and human organogenesis. Our analyses showed that the genes in the up-regulated and down-regulated gene clusters were mostly conserved during mouse and human organogenesis and are likely responsible for the similarities between mouse and human organogenesis. On the other hand, the genes in the arch-up regulated gene cluster, which are less conserved between mouse and human organogenesis, and those in the arch-down regulated gene cluster, which is unique to mouse organogenesis, appear to be critical for the divergence between mouse and human embryogenesis.

### A molecular interaction network governing mouse organogenesis

**Figure 6:**
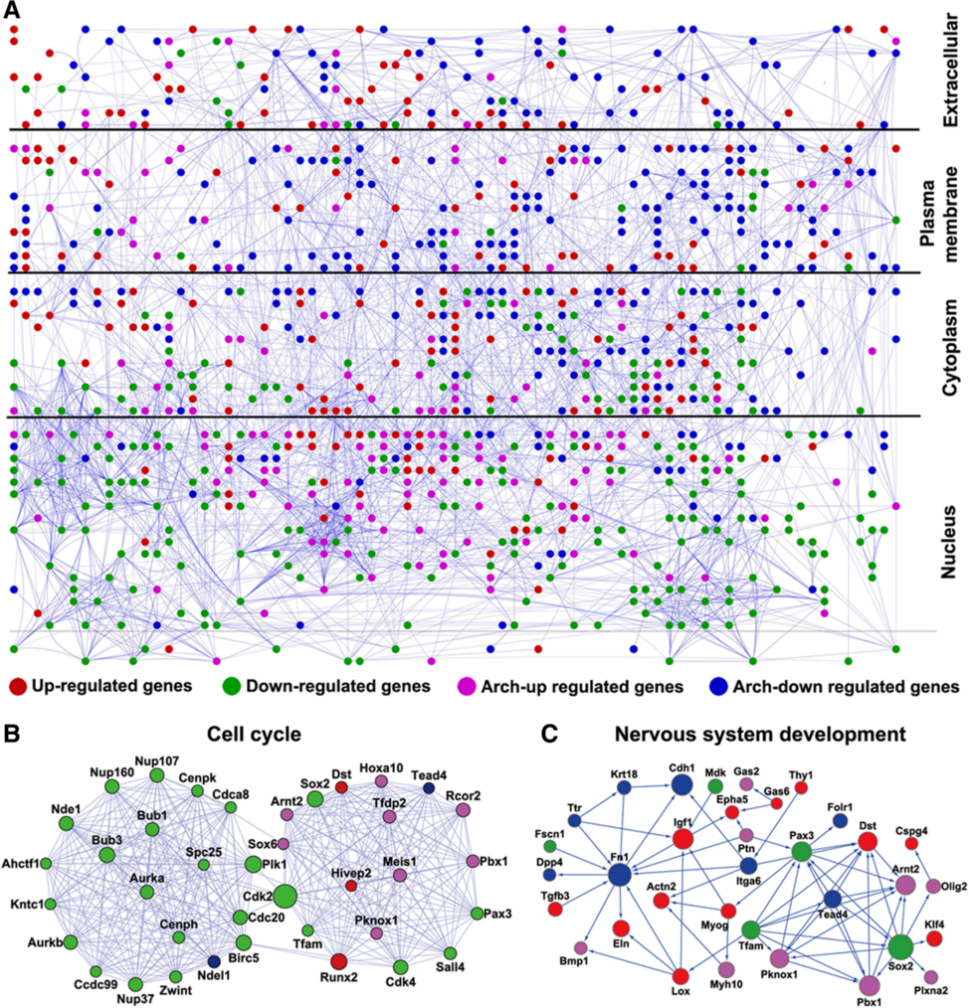
**A molecular interaction network during mouse organogenesis. (A)** The 1000 most significantly regulated genes among the 4 major patterns shown in Figure 4 during mouse organogenesis were analyzed by using the STRING database to obtain a protein interaction network. Each line between any two proteins (dots) indicates an interaction. The genes are grouped into 4 categories based on their cellular locations. The color indicates the gene regulation patterns as in Figure 4. **(B)** The cell cycle sub-network showing that many interacting proteins have similar expression profiles: most genes on the left circle are down-regulated while those on the left are arch-up regulated. The size of each dot is proportional to the number of interactions the protein has within the network. **(C)** The neurogenesis genes subnetwork showing that the arch-down genes may function to bridge interactions among the proteins in different regulation groups in the network.

Embryogenesis involves complex spatiotemporal interactions among diverse groups of genes. To determine if and how the genes that are regulated during mouse organogenesis interact with each other, we used the STRING database and the Cytoscape visualizing tool to characterize the signaling and regulatory interactions among the regulated genes [50,51]. As show in Figure 6A, all proteins encoded by the regulated genes were color-coded based on their expression pattern. Among four groups of genes, proteins encoded by genes in the up-regulated, down-regulated and the arch-up regulated have more interactions among themselves than with proteins outside their own groups (p < 0.01). To investigate the details of this protein interaction network, we analyzed densely connected regions of the network. Figure 6B shows a sub-network with proteins closely related to cell cycle and mitosis (p = 0) as detected by MCODE analysis [52]. Most proteins in this subnetwork were downregulated, as expected from the analysis of the downregulated group above. Importantly, the interaction network showed that among the cell cycle related genes, the downregualted ones interacted with each other while the small fraction of upregulated cell cycle related genes were clustered into a different subnetwork, demonstrating that genes functioned together were coordinated regulated.

The interaction analysis also allowed us to assign the cellular localizations of the encoded proteins (Figure 6A). Interestingly, the arch-down regulated genes encoded disproportionally proteins located on the plasma membrane and they had more interactions with proteins encoded by genes in the other regulation groups than with themselves (Figure 6A). Our analysis above suggests that the arch-down regulated genes likely play important roles in determining the species-specific development in mouse. The interaction pattern of the downregulated group indicates that the arch-down genes may function to bridge interactions among genes in different regulation groups. As an example, by using the jActive-module of Cytoscape [53], we identified a subnetwork that included all four groups of expression patterns shown in Figure 4 and were significantly enriched with genes related to nervous system development (p = 0) (Figure 6C), showing clearly that several proteins of the arch-down regulated group had extensive interactions with proteins encoded by genes in the other regulation groups (Figure 6C).

### Efficiency of gene utilization as a potential developmental strategy in different organisms

Evolution from lower to higher species can be exhibited in different aspects such as body morphology and size, organ functions, tissue structures, as well as genomic organizations, etc. [54]. At the genome level, the C-value enigma indicates that the size of genome does not represent the evolutionary order of different species [55,56]. Based on our analyses of the transcriptome during mouse and human organogenesis, we wonder if transcriptome regulation may be an indicator of evolution. Considering efficiency as a selective pressure in evolution, different species may need alter the expression of different number of genes for a similar developmental process. To investigate this possibility, we focused on the mouse developmental period from TS13 to TS27 and the corresponding human developmental period, i.e., from week 4 to week 9, and analyzed the percentages of genes expressed or regulated. The total number of genes expressed in human [18] and mouse (this study) were 28,761 and 27,966 (Table 1), accounting for 57.4% and 71.6% of the total genes identified in both species, respectively, which was statistically different (p = 0). Thus, a higher percentage of mouse genes than human genes participated in development. Furthermore, we found that the number of genes that were regulated during human and mouse organogenesis, i.e., from stage TS13 to TS27 for mouse and week 4 to week 9 for human, were 5,358 and 8,521, which accounted for 10.7% and 21.8% of the total genes in human and mouse, respectively. Again, the regulated genes as a percentage of total genes needed for organogenesis were significantly different between the two species (p = 0), i.e., mouse needs a higher percentage of regulated genes than human does for organogenesis. Taking together, we suggest that the more evolutionarily advanced species, e.g., human, the more efficient it is in gene utilization for development. To further test this possibility, we analyzed the transcriptome data for the organogenesis of zebrafish [57], a less advanced species compared to mouse (Table 1). We found that as many as 68.9% genes participated during zebrafish organogenesis, significantly higher than that of mouse (p = 0). Thus, the evolutionary strategy for zebrafish appears to be using more genes than those during mouse development (p = 0), consistent with the findings from the comparison of the mouse and human transcriptome. Thus, the transcriptome activity during different periods of embryogenesis may serve as a new criterion to evaluate evolutionary strategy in different species. Clearly additional data from deliberately designed studies are needed to further support and validate this hypothesis.

**Table 1:** Comparison of transcriptome activity during organogenesis among different species

	**Number of differentially expressed genes**	**Number of expected differentially expressed genes**	**Number of expressed genes**	**Number of expected expressed genes**	**Number of genes on the microarray**
**Human**[18]	5358	—	28761	—	50093
**Mouse**	8521	6077	27857	24789	39014
**Zebrafish**[57]	15094	8488	—	—	21893

## Conclusions

We have reported here the first genome-wide gene expression analysis of the entire mouse embryogenesis. Our data indicate that the GO categories or signaling processes that are significantly regulated during development correlate well with the developmental changes taking place at the particular stages. Furthermore, our comparative analyses suggest that a unique group of genes with a distinct regulation pattern during mouse organogenesis that is absent during human development underlies the developmental divergences between human and mouse, while the majority of the genes in the three other major temporal regulation groups are conserved between mouse and human, consistent with the general similarity of mouse and human development. Given our earlier observation that genes function in a given process tends to be developmentally co-regulated during organogenesis [18,25], our microarray data here should help to identify genes associated with mouse development and/or infer the developmental functions of unknown genes. Finally, our findings suggest that the complexity of gene regulation during development may serve as an evolutionary strategy in vertebrates.

## Methods

### Ethics statement

All animal experiments were approved by the Animal Research Ethics Board of Wuhan University in China and were in compliance with institutional guidelines on the care of experimental animals.

### RNA preparation

All mice used were obtained from ABSL-3 lab, Wuhan University. F1 females were superovulated by injecting first 10 IU PMSG and then 10 IU hCG 48 ~ 50 h later. Oocytes were collected 14 ~ 16 h after hCG treatment. 25 ~ 50 oocytes were obtained from each successfully supervulated mice. Each biological replicate had 500 oocytes or more. To collect fertilized eggs, females were mated with healthy F1 males and fertilized eggs were collected at 18 h after hCG. Each replicate had 500 eggs or more. The ages of the post-implantation embryos were carefully determined according to the standard protocol (Additional file 1A). For each biological replicate more than 20 embryos were dissected from different F1 females. At each stage 3 replicates were used for microarray analysis and another was kept for RT-PCR validation of the microarray results. All samples were homogenized in Trizol Reagent (Invitogen, USA) for further RNA isolation.

### Real-time quantitative RT-PCR

This was done with the primers for indicated genes (Additional file 16) and the SYBR^®^ qPCR Mix (Toyobo, Japan) on an ABI PRISM 7500 and analyzed using the Sequence Detection System 2.0 software.

### Microarray analyses

The gene expression profiles of the mouse samples were performed by using the Affymetrix Mouse Genome 430 2.0 GeneChip microarrays (Affymetrix, Santa Clara, CA) according to the manufacturer’s protocol. The raw expression data were normalized using Affymetrix Microarray Suite 5.0 (MAS 5.0) with quantile normalization. The normalized data for all arrays have been deposited in the Gene Expression Omnibus (GEO) at the NCBI [58] and are accessible through GEO Series accession number GSE39897 (http://www.ncbi.nlm.nih.gov/geo/query/acc.cgi?acc=GSE39897). The Pearson’s correlation coefficient was calculated to show a high degree of reproducibility of among the replicas (Additional file 1B). A transcript was scored as “detected” or “expressed” if significant signal was detected on two or three of the individual microarrays for the three independent replicas [28].

### Bioinformatics analysis of developmentally regulated genes

*T*-test was used to detect differentially expressed genes between adjacent developmental stages with a p value cutoff of 0.05. Genes that exhibit expression differences only in one pair of developmental time points were defined as “unique differentially expressed genes”. The MaSigPro [34] procedure was employed to detect the transcripts exhibiting consistent changes within the triplicates as well as differential expression across whole developmental stages or organogenesis.

The major gene expression patterns were identified by using the Serial Expression Analysis (SEA) tools [59]. For detection of significantly changed transcripts, R-Squared was set to 0.8 when using the MaSigPro method supplied in the SEA online system. These differentially expressed genes were then clustered via the hierarchical clustering method to explore their major expression trends. All differentially expressed gene sets were then subjected to the DAVID web program [35] to identify enriched biological themes and quantify the function categories. Function categories was consider significant only if the p < 0.001, enrichment fold > 2 and FDR < 0.001.

### Comparisons of gene expression data during organogenesis between mouse, human And Zebrafish

The transcriptome data of the organogenesis embryos during organogenesis (CS9-23, which resembles the mouse organogenesis period of TS13-TS27) and zebrafish embryos during organogenesis were obtained from NCBI GEO [GSE15744 and 24840, respectively] [18,57]. The up-regulated, down-regulated and arch-up-regulated clusters of mouse genes were used for the comparison with relevant clusters during human organogenesis. The significance of overlaps for each comparison was evaluated using the Fisher’s exact test. The human homologs of the mouse genes were obtained by using MGI database homology data [2].

The mouse, human, and zebrafish array data were subjected to the same normalization method and differential gene detection method (1-way ANOVA, p < 0.05) before comparing the number of expressed and differentially expressed transcripts among three species.

Chi-square test was used to quantify that the numbers of regulated genes needs for organogenesis were significantly different in three species.

### Construction of the mouse organogenesis subnetworks

Genes from four regulated clusters during mouse organogenesis were subjected to the STRING (medium confidence, ≥0.4) analysis to obtain their interaction network [50]. The Cytoscape plug-in Cerebral was used to visualize the subnetwork, which is configured based on subcellular localization information of genes [51]. These localization data were obtained from the online DAVID GO Terms including the “nucleus” (GO: 0005634), “cytoplasm” (GO: 0005737), “plasma membrane” (GO: 0005886), and “extracellular region” (GO: 0005576). The MCODE plug-in was used to detect densely connected regions in large protein-protein interaction networks that may be related to molecular complexes [52]. The jActive-module plug-in was used to identify connected regions of the network that show significant changes in expression over particular subsets of expression conditions [53]. Chi-square test was used to quantify that the genes have more interactions among the genes within their own groups than with genes outside their own groups.

### Accession codes

The data from this study have been submitted to the NCBI Gene Expression Omnibus (GEO) (http://www.ncbi.nlm.nih.gov/geo/) under accession number [GSE39897].

## Abbreviations

CS: Carnegie stage; TS: Theiler stage; GO: Gene ontology; MGD: Mouse genome database; GEO: Gene expression omnibus; MGI: Mouse genome informatics; SEA: Serial expression analysis.

## Competing interest

The authors have declared that no competing interests exist.

## Authors’ contributions

LX, MXG, YBS and WXL conceived and designed the experiments. LX, JYC, JM and MXG performed the experiments. LX, JYC, JM, ZH, MXG, LF, YBS and WXL analyzed the data. LX, ZH, LF, YBS and WXL wrote the manuscript. All authors read and approved the final manuscript.

## Supplementary Material

Additional file 1 Is a figure showing information concerning microarray sample. Click here for file

Additional file 2 Lists all expressed genes with their stages of expression and the stages, if any, when the gene was uniquely regulated. Click here for file

Additional file 3 Is a figure showing 20 clusters for 11,458 regulated genes during mouse embryogenesis. Click here for file

Additional file 4 Is a table listing the 11,458 genes regulated during mouse embryogenesis from egg to TS27. Click here for file

Additional file 5 **Is a table listing enriched GO categories for the Cluster I in Additional file **4**.** Click here for file

Additional file 6 **Is a table listing enriched GO categories for the Cluster II in Additional file**4. Click here for file

Additional file 7 **Is a table listing enriched GO categories for the Cluster III in Additional file **4. Click here for file

Additional file 8 **Is a table listing enriched GO categories for the Cluster IV in Additional file **4. Click here for file

Additional file 9 **Is a table listing enriched GO categories for the Cluster V in Additional file **4. Click here for file

Additional file 10 **Is a table listing enriched GO categories for the Cluster VI in Additional file **4. Click here for file

Additional file 11 Is a table listing the 8,521 genes regulated during mouse organogenesis from TS13 to TS27. Click here for file

Additional file 12 **Is a table listing enriched GO categories for the UP group in Additional file **11. Click here for file

Additional file 13 **Is a table listing enriched GO categories for the DOWN group in Additional file **11. Click here for file

Additional file 14 **Is a table listing enriched GO categories for the ARCH-DOWN group in Additional file **11. Click here for file

Additional file 15 **Is a table listing enriched GO categories for the ARCH-UP group in Additional file **10. Click here for file

Additional file 16 **Is a table listing primers for real-time quantitative RT-PCR shown in Additional file **1. Click here for file
