# Supplementary material for: Global expression profiling reveals genetic programs underlying the developmental divergence between mouse and human embryogenesis
Source: BMC Genomics. 2013 Aug 20;14:568. doi: 10.1186/1471-2164-14-568 (PMC3924405; doi:10.1186/1471-2164-14-568)
Supplement: Additional file 1 — Is a figure showing information concerning microarray sample. [file 1471-2164-14-568-S1.pdf]

A

Information concerning mouse embryo collection and staging

Dpc	Staging Criteria[§]	Equivalent Theiler Stage *(mouse)*[†]	Equivalent Carnegie Stage *(Human)*[†]
Oocyte	Mature eggs	/	/
E0.5	Fertilized eggs	TS01	CS01
E 4	Free blastocyst	TS05	CS03
E6.5	Advanced endometrial reaction	TS09	CS06
E7.5	Neural plate, presomite stage	TS11	CS08
E8.5	Turning of the embryos, 8-12somites	TS13	CS10
E10	Closure of posterior neurepore; hind limb bud and tail bud	TS16	CS13
E11.5	Lens vesicle completely separated from surface	TS19	CS16
E13	Anterior footplate indented, marked pinna	TS21	CS18
E14	Fingers separate distally	TS22	CS20
E15	Toes separate	TS23	CS21~22
E17	Fingers and toes joined together	TS25	Fetal period
Newborn	Newborn mouse	TS27	

[§] reference: Theiler, The house mouse. Springer-Verlag, NY (1972)
[†] resource: Edinburgh Human Developmental Anatomy;
O'Rahilly, Early human development and the chief source of information on staged human embryos. Eur. J. Obstet. Gynec. Reprod. Biol. 9 p273 (1979)

B

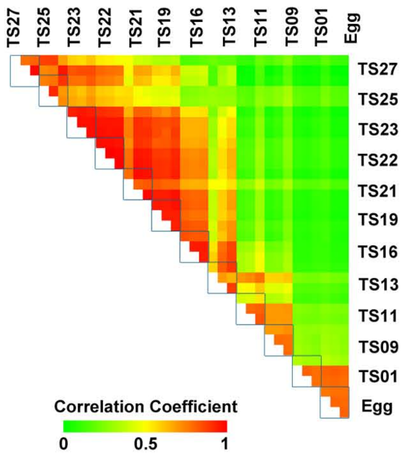

C

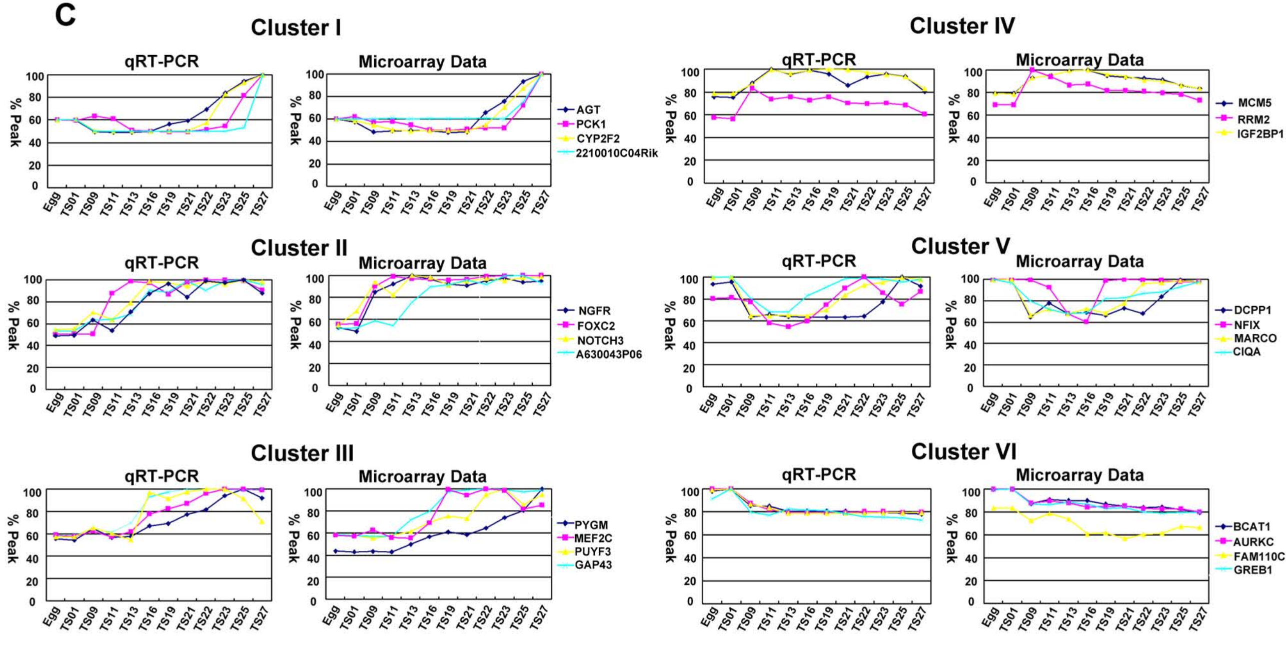

Additional file 1 (A) Information concerning mouse embryo collection and staging.

(B) Pair-wise correlation analyses of transcriptome profiles of microarray samples. The colors relate to Pearson's correlation coefficient values, with colors indicating positive (red) or negative (green) correlations among the samples. Note that in general, embryos of closedly related stages had similar overall gene expression profiles (red, near the diagonal line) compared to those further apart (green, near the base of the triangle), consistent with the staging of the embryos.

(C) Validation of microarray data by quantitative RT-PCR on genes selected from the six developmental clusters as shown in Figure 3. Experiments were performed with independent RNA samples. Data are represented as mean values. Note the temporal regulation pattern for each gene as determined by RT-PCR agreed with that determined by microarray.
