# Supplementary material for: Global expression profiling reveals genetic programs underlying the developmental divergence between mouse and human embryogenesis
Source: BMC Genomics. 2013 Aug 20;14:568. doi: 10.1186/1471-2164-14-568 (PMC3924405; doi:10.1186/1471-2164-14-568)
Supplement: Additional file 3 — Is a figure showing 20 clusters for 11,458 regulated genes during mouse embryogenesis. [file 1471-2164-14-568-S3.pdf]

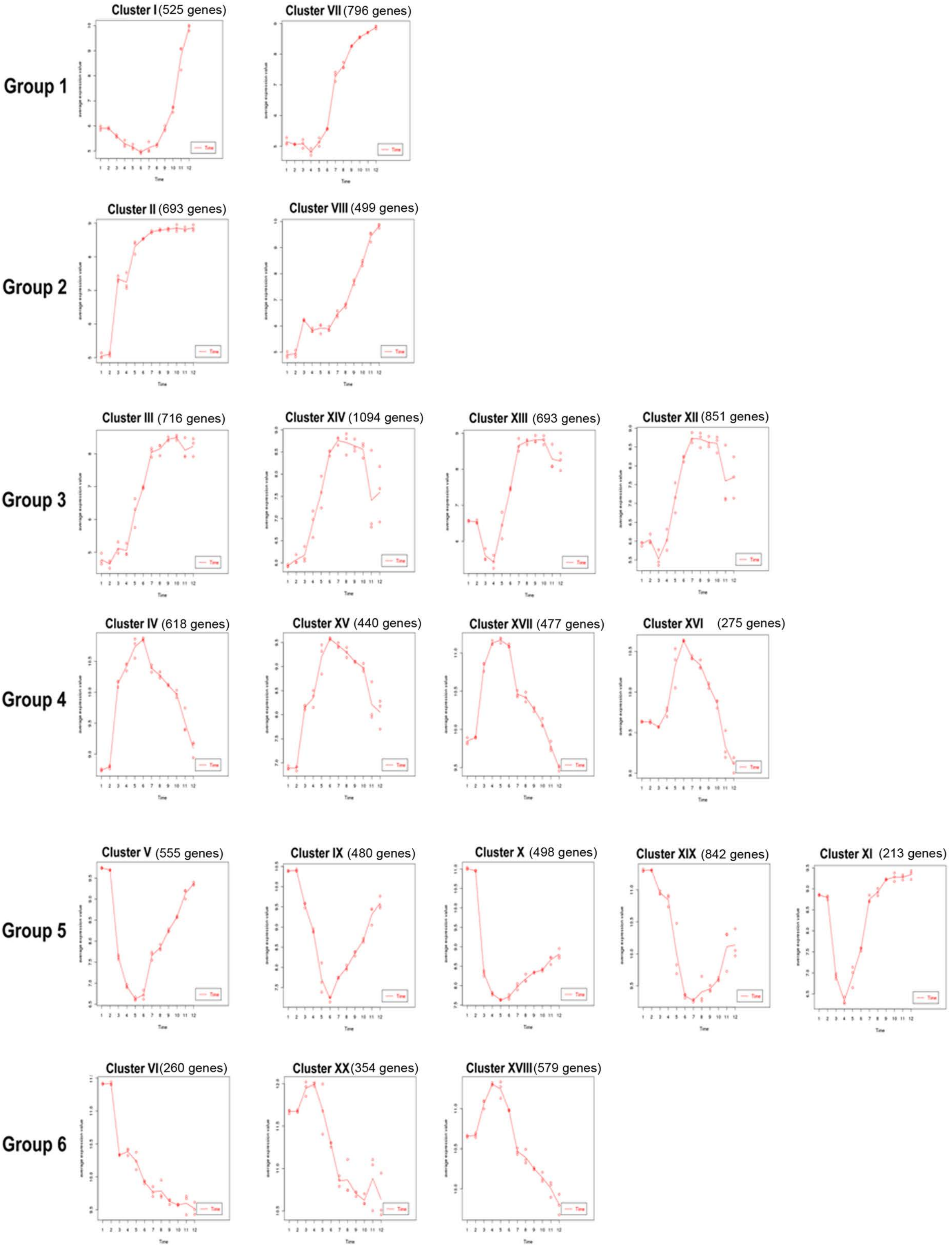
Group 1
Cluster I (525 genes)
Cluster VII (796 genes)
Group 2
Cluster II (693 genes)
Cluster VIII (499 genes)
Group 3
Cluster III (716 genes)
Cluster XIV (1094 genes)
Cluster XIII (693 genes)
Cluster XII (851 genes)
Group 4
Cluster IV (618 genes)
Cluster XV (440 genes)
Cluster XVII (477 genes)
Cluster XVI (275 genes)
Group 5
Cluster V (555 genes)
Cluster IX (480 genes)
Cluster X (498 genes)
Cluster XIX (842 genes)
Cluster XI (213 genes)
Group 6
Cluster VI (260 genes)
Cluster XX (354 genes)
Cluster XVIII (579 genes)

Additional file 3 20 clusters for 11,458 regulated genes during mouse embryogenesis. Cluster analysis of 11,458 regulated genes was performed using the Serial Expression Analysis tools. Then all the 20 clusters were divided into six groups of related clusters for further analysis. One representative cluster, cluster I to VI, was chosen as a representative for group 1 to 6, respectively, and shown on the left.
