# Supplementary material for: Global expression profiling reveals genetic programs underlying the developmental divergence between mouse and human embryogenesis
Source: BMC Genomics. 2013 Aug 20;14:568. doi: 10.1186/1471-2164-14-568 (PMC3924405; doi:10.1186/1471-2164-14-568)
Supplement: Additional file 16 — Is a table listing primers for real-time quantitative RT-PCR shown in Additional file 1. [file 1471-2164-14-568-S16.pdf]

Additional file 16 Primers for real-time quantitative RT-PCR shown in Additional file 1.

Gene Name	Forward/Reverse Sequence	Gene Number
Agt	F: TCGAACTCAAAGCAGGAGA<br>R: TCGTAGATGGCGAACAGG	NM_007428
Pck1	F: TGCTCCAGCTTTGAGATA<br>R: GAGACTATGCGGTGATTT	NM_011044
Cyp2f2	F: GCCTGCCACGTATCAGTA<br>R: GCGTATAAGACGGGTATTTC	NM_007817
2210010C04Rik	F: ACCCAGGCAAGATCACTA<br>R: TCTGAGCACAGCCATAAC	NM_023333
Ngfr	F: ACAGACTGACTGCCATCCC<br>R: TGCAGATCGAGGTCCATAA	NM_033217
Foxc2	F: CCAACTGTTACTGCCAAAT<br>R: AAGCCATGCACTTCCTAA	NM_013519
Notch3	F: GCTCAAAGCCTCAACTCC<br>R: CCCACCTTCTGCCCTGTA	NM_008716
A630043P06	F: GCATCCCTCTGGGAACTT<br>R: TCAAAAGGATAGCACCAA	NM_133643
Pygm	F: AATGCCAGGACAAAGTCA<br>R: CGAGAAGGTTCAACACCC	NM_011224
Mef2c	F: CGGTAATTGTAGGAACACGC<br>R: GTGCCATACGCCAATGATAT	NM_001170537
Rufy3	F: TGGTATGGATTCCTCGTT<br>R: CATCACTAAGGCAGAAATAA	NM_027530
Gap43	F: CGACAGGATGAGGGTAAAG<br>R: AGACAGGGTTCAGGTGGG	NM_008083
Mcm5	F: ATCCAGAGCACGCTATCC<br>R: GGTGGTGGCAGCATTTCA	NM_008566
Rrm2	F: GTCAGGCGATTAAACTAT<br>R: TGACACTATTAGGGCAAA	NM_009104
Igf2bp1	F: CGGACTTGGCTAGTTTGT<br>R: ATGAGATTGCTTTGTGGC	NM_009951
Dcpp1	F: CAACAAGGACGGTCAAGT<br>R: GTTAAAGCCGAAGGAGTG	NM_019910
Nfix	F: GACCTTTATCTGGCTTACTTTG<br>R: TTTGATGTCCGCATCTCC	NM_001081982
Marco	F: AGGCGAATCTTTCCAACG<br>R: CGCATCATTATTATCCCAAT	NM_010766
C1qa	F: GCACTGAAGCCGACAGCA<br>R: CGCAGGAGATGGCAGGAT	NM_007572

Bcat1	F: TGTCTGAGGCTGGTTGTC<br>R: ACTATGTGCTGGGCTTTG	NM_001024468
Aurkc	F: GTGGGACTCTGGACTACTTG<br>R: ATGTCTCACTGGAGGTGC	NM_001080965
Fam110c	F: GTCCTTCCGTTAGCTCTTTA<br>R: CAGGCAATTCCACTGTATGT	NM_027828
Greb1	F: GAGCTATCTTGAAGTCCGTTAT<br>R: TCATGGTCACAGGCATCG	NM_015764
